# Parliament and Publications

**Published:** 1910-12-31

**Authors:** 


### Parliament and Publications.

DUTIES OF MEDICAL OFFICERS OF HEALTH.

The Local Government Board have made some new regulations relating to medical officers of health. One of these, which comes into force on January 1, 1911, requires the medical officer to send to the Board weekly a list of cases of infectious disease notified in his district and a duplicate of the list to the medical officer of health for the county. The medical officer is also required to report to the Board forthwith any case of plague, cholera, or smallpox brought to his knowledge, but it will no longer be necessary to report to the Board the cases in which he advises the closure of any school in his district. New conditions are prescribed as to tenure of office, and these will come into force on April 1, 1911. An officer appointed for a specified term will continue to hold office from year to year after the expiration of that term, and no further approval by the Board will be necessary unless the terms of the appointment are altered. If the Council wish to dispense with the services of an officer at the end of any year they must give him three months' notice of their intention. (Order No. 56,217.)

